# Supplementary material for: Single-catalyst high-weight% hydrogen storage in an N-heterocycle synthesized from lignin hydrogenolysis products and ammonia
Source: Nat Commun. 2016 Oct 20;7:13201. doi: 10.1038/ncomms13201 (PMC5080437; doi:10.1038/ncomms13201)
Supplement: Supplementary Information — Supplementary Figures 1-20, Supplementary Tables 1-5, Supplementary Note 1, Supplementary Methods and Supplementary References [file ncomms13201-s1.pdf]

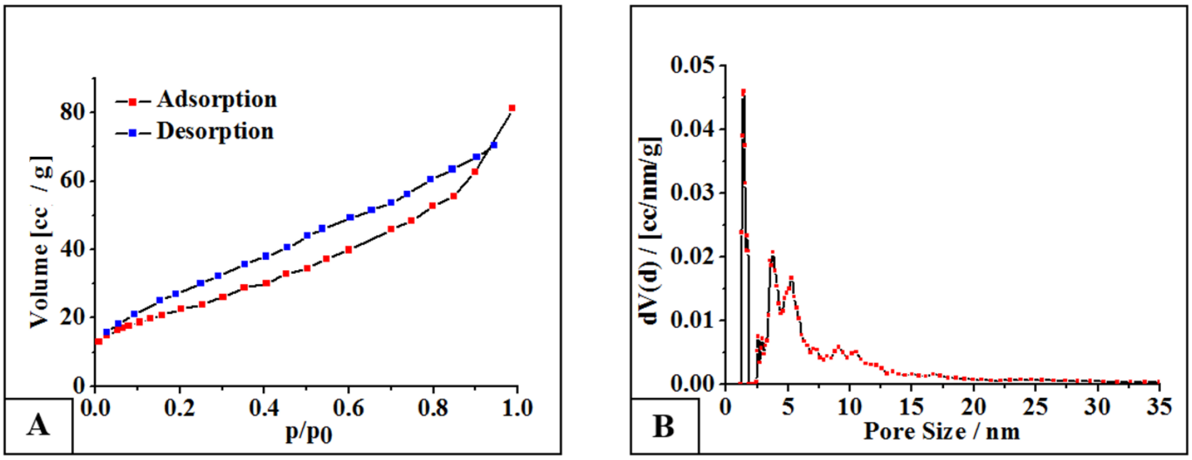

Supplementary Figure 1: N_2 sorption analysis of the Pd_2Ru@SiCN catalyst; (A) Nitrogen sorption isotherme; **(B)** Calculated pore size distribution.

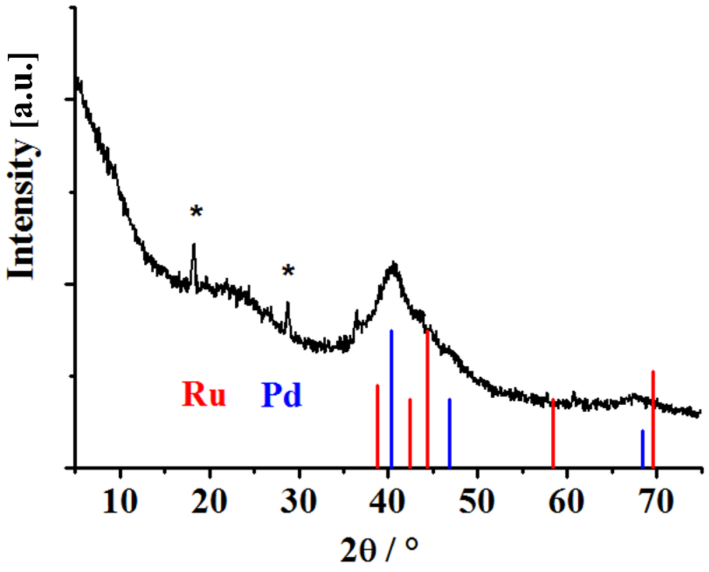

Supplementary Figure 2: Powder XRD analysis of the Pd_2Ru@SiCN catalyst; (red: Reflexes of hexagonal crystalline ruthenium; reference card: 00-001-1253; blue: Reflexes of cubic crystalline palladium; reference card: 00-001-1201; * SiO_2).

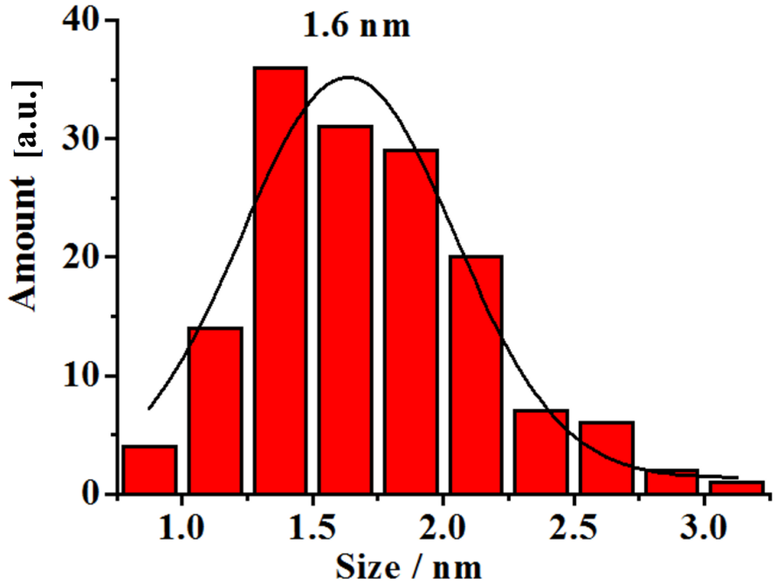

Supplementary Figure 3: Particle size distribution of the palladium nanoparticles.

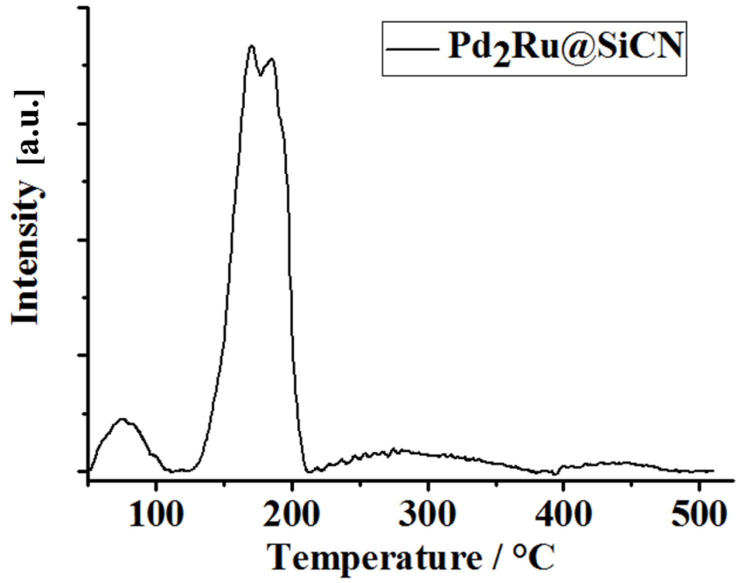

Supplementary Figure 4: TPR analysis of the Pd_2Ru@SiCN catalyst.

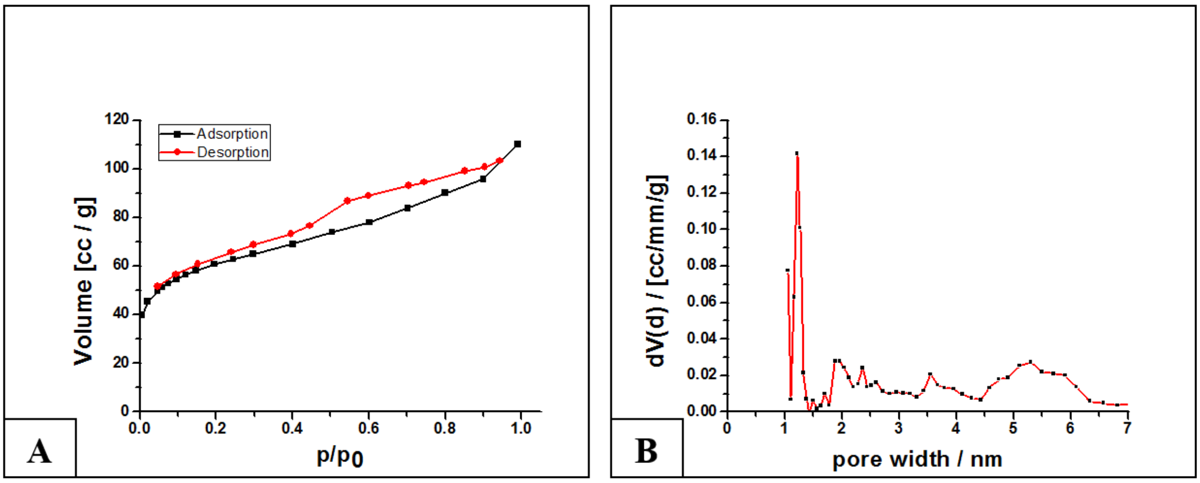

Supplementary Figure 5: N_2 sorption analysis of the Ru@SiCN catalyst. (**A**) Nitrogen sorption isotherme. (**B**) Calculated pore size distribution.

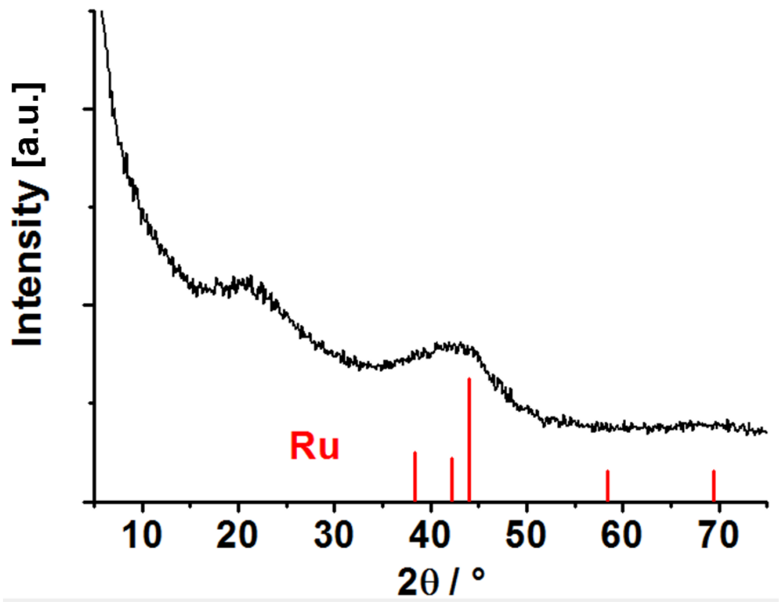

Supplementary Figure 6: Powder XRD analysis of the Ru@SiCN catalyst; (red: Reflexes of hexagonal crystalline ruthenium; reference card: 00-001-1253).

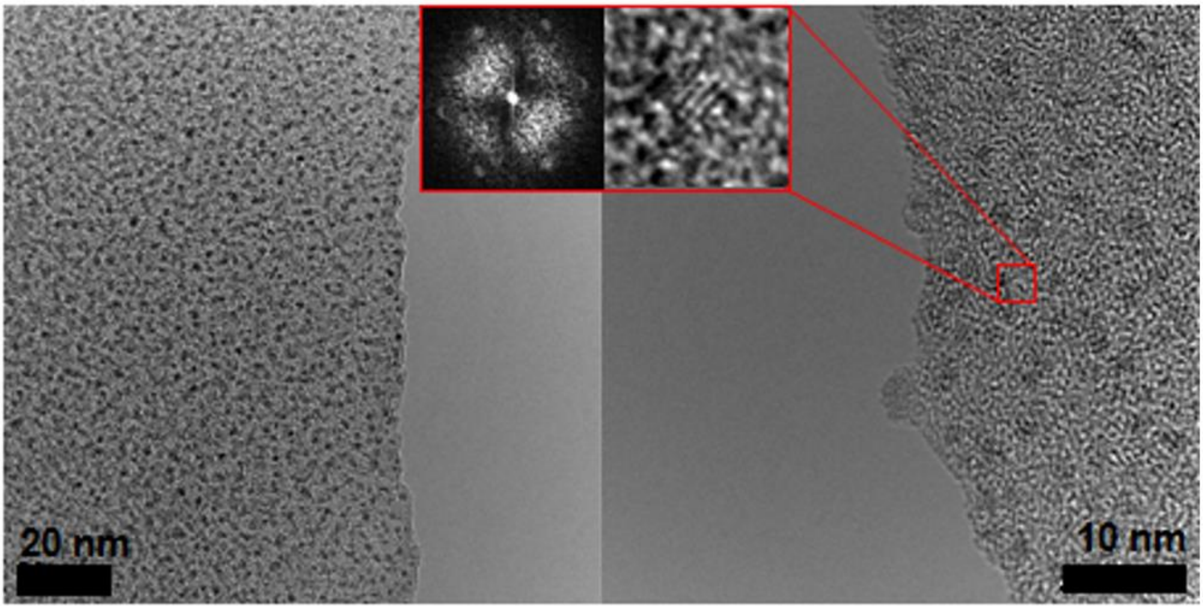

Supplementary Figure 7: HR-TEM analysis of the Ru@SiCN catalyst; left & right: HR-TEM picture. middle: Magnification of one Ru nanoparticle with the corresponding FFT.

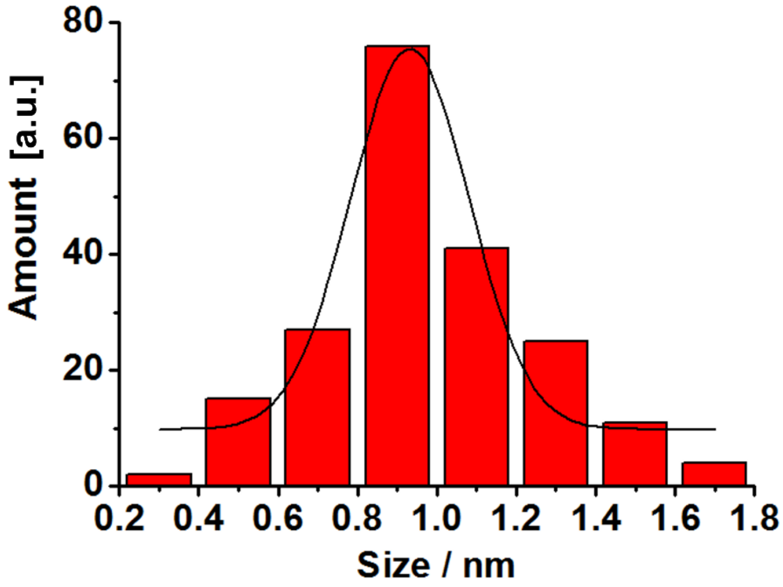

Supplementary Figure 8: Particle size distribution of the ruthenium nanoparticles.

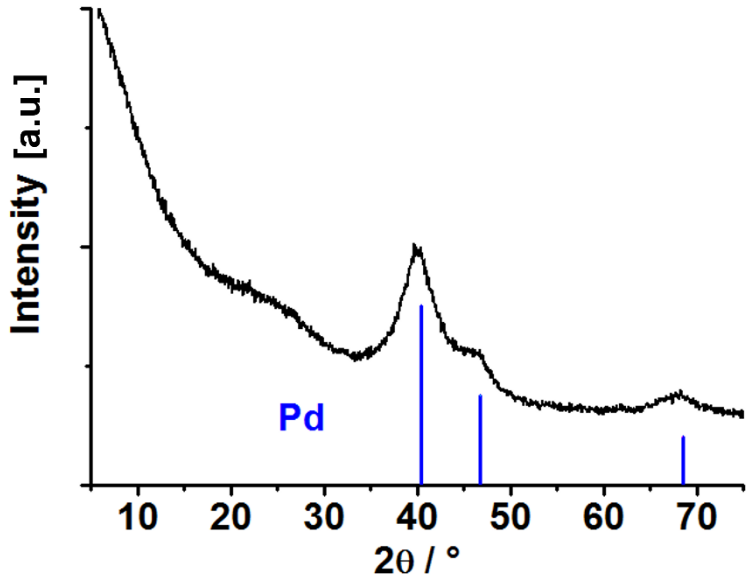

Supplementary Figure 9: Powder XRD analysis of the Pd@SiCN catalyst; (blue: Reflexes of cubic crystalline palladium; reference card: 00-001-1201).

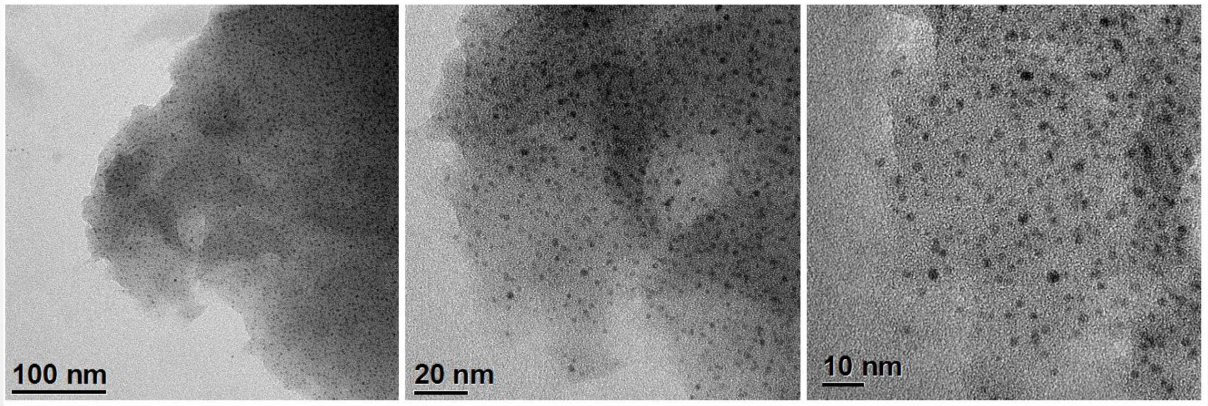

Supplementary Figure 10: TEM analysis of the Pd@SiCN catalyst.

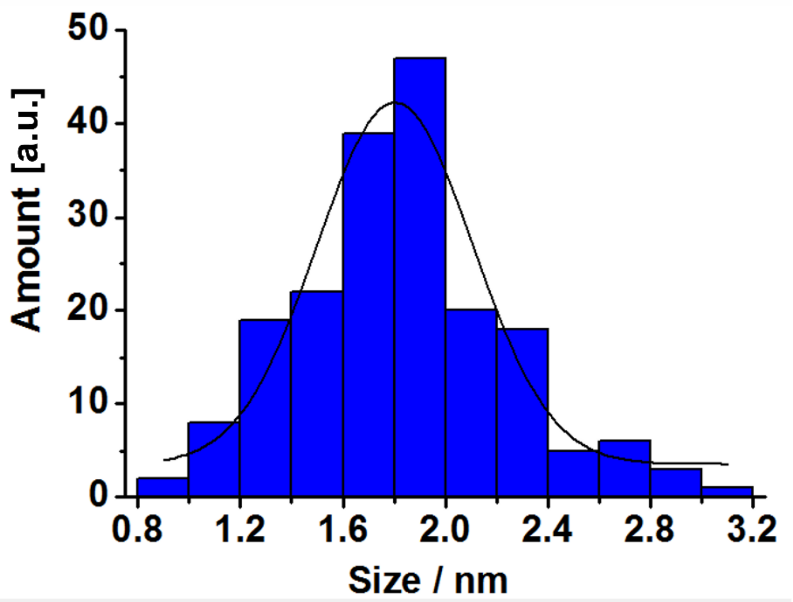

Supplementary Figure 11: Particle size distribution of the palladium nanoparticles.

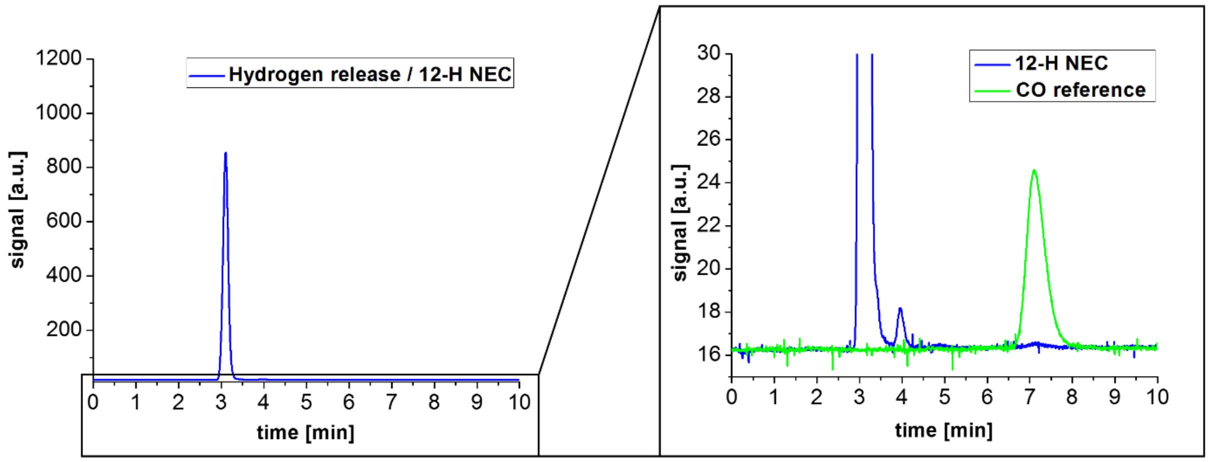

Supplementary Figure 12: GC measurements of the released gas; Left: Blue graph verifies the release of hydrogen while dehydrogenation of 12-H NEC. **Right:** Comparison with a CO reference sample confirms that no CO is produced while dehydrogenation process. Small amounts of atmospheric nitrogen are unavoidable by manual injection.

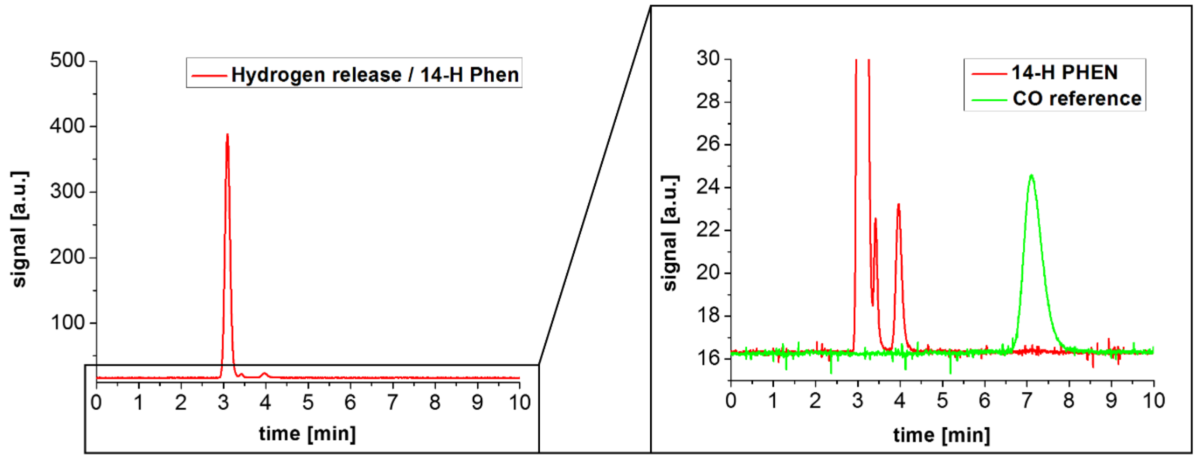

Supplementary Figure 13: GC measurements of the released gas; Left: Red graph verifies the release of hydrogen while dehydrogenation of 14-H Phen. **Right:** Comparison with a CO reference sample confirms that no CO is produced while dehydrogenation process. Small amounts of atmospheric nitrogen as well as oxygen are nearly unavoidable by manual injection.

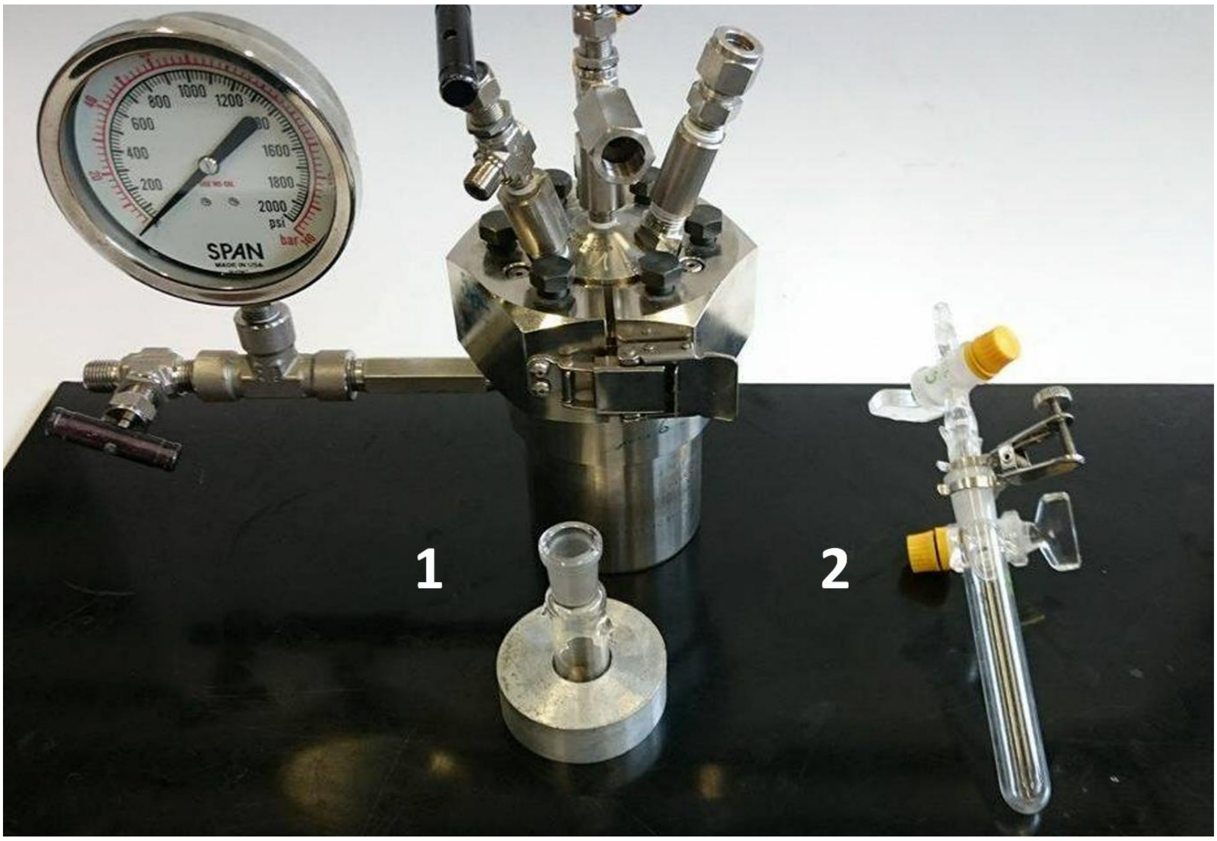

Supplementary Figure 14: Devices used for reversible hydrogen storage: 1) Hydrogenation was accomplished in a 10 ml reaction vial, placed in a 300 ml steel autocalve (Parr Instrument). Dehydrogenation was performed in a 10 ml Schlenk tube. For hydrogen release experiments, the Schlenk tube was linked with a flexible tube and the released gas was collected by water column.

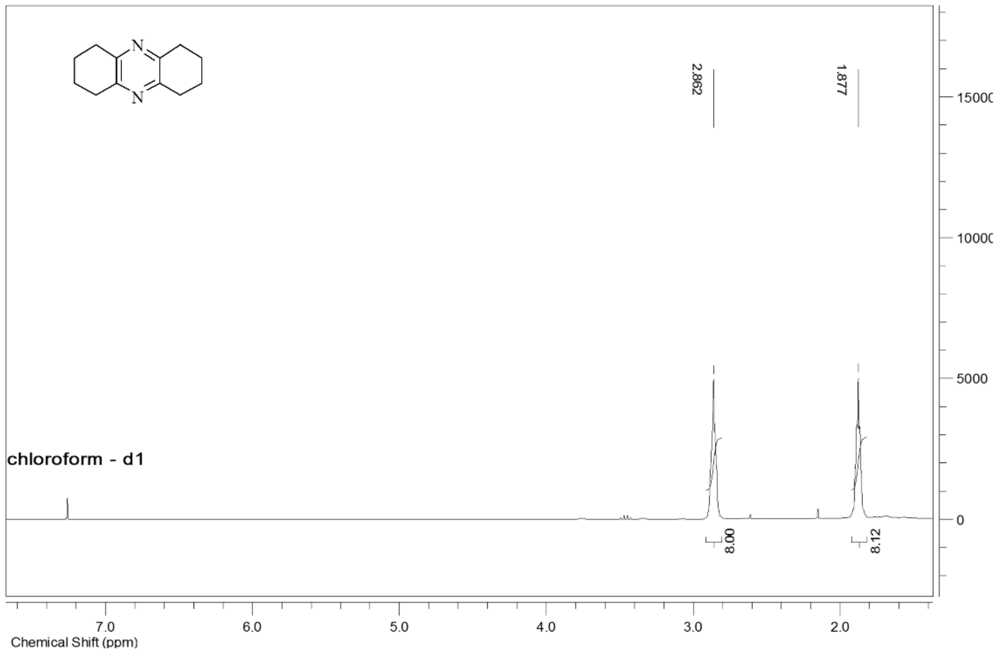

Supplementary Figure 15: ^{1}H-NMR Spectra of 1,2,3,4,6,7,8,9-octahydrophenazine.

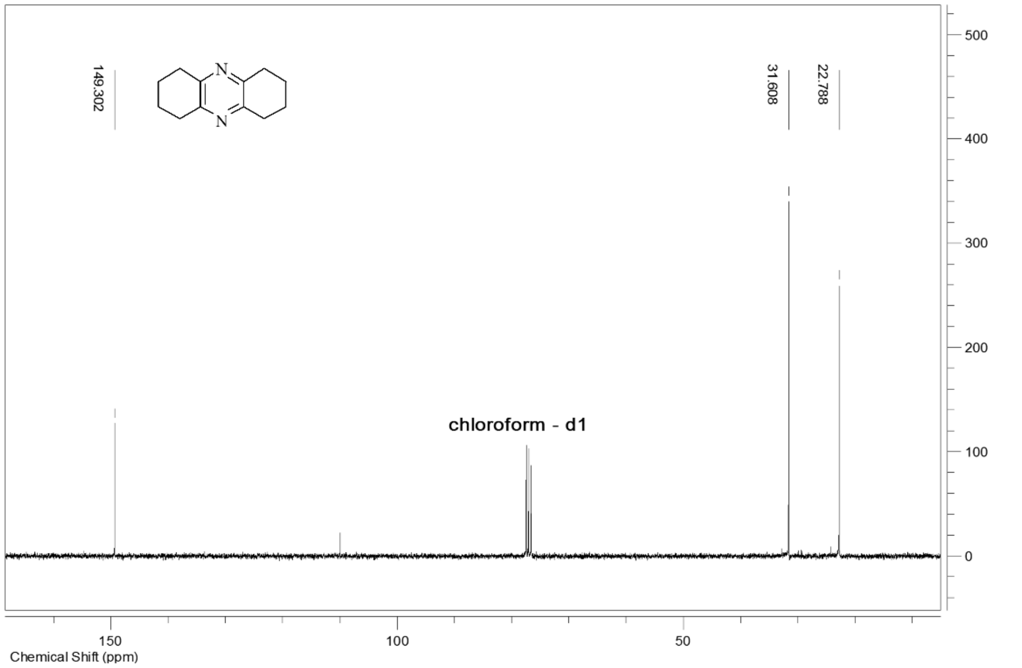

Supplementary Figure 16: ^{13}C-NMR Spectra of 1,2,3,4,6,7,8,9-octahydrophenazine.

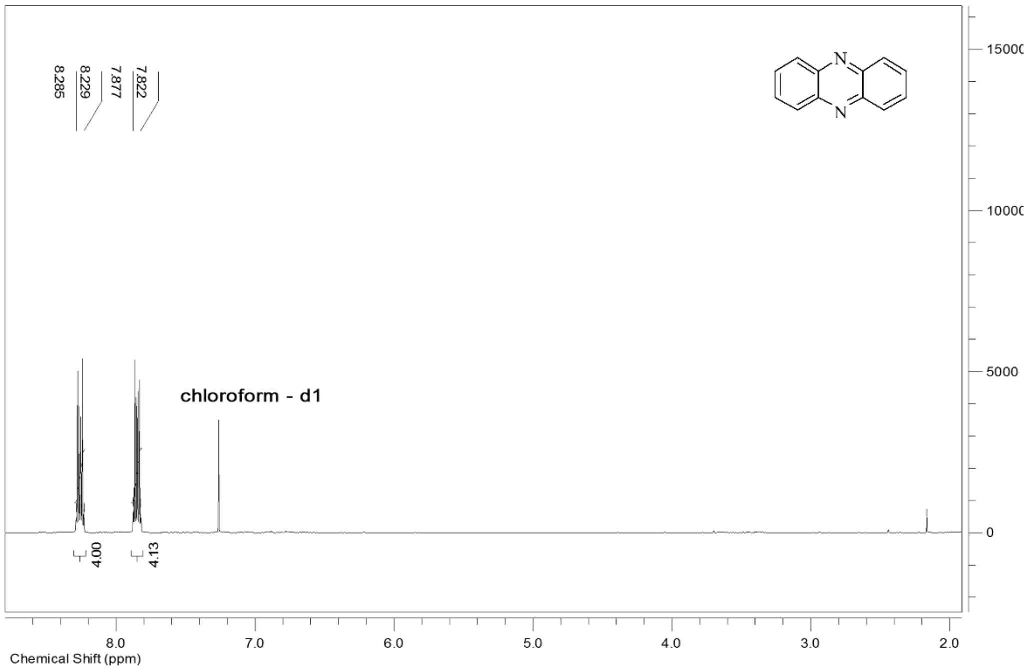

Supplementary Figure 17: ^{1}H-NMR Spectra of phenazine.

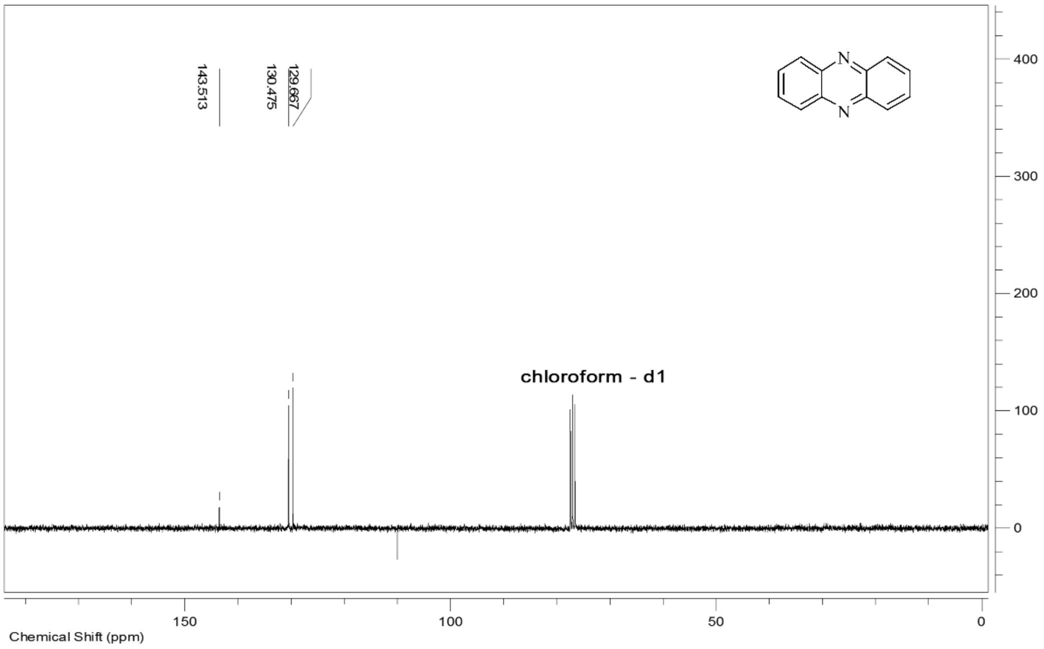

Supplementary Figure 18: ^{13}C-NMR Spectra of phenazine.

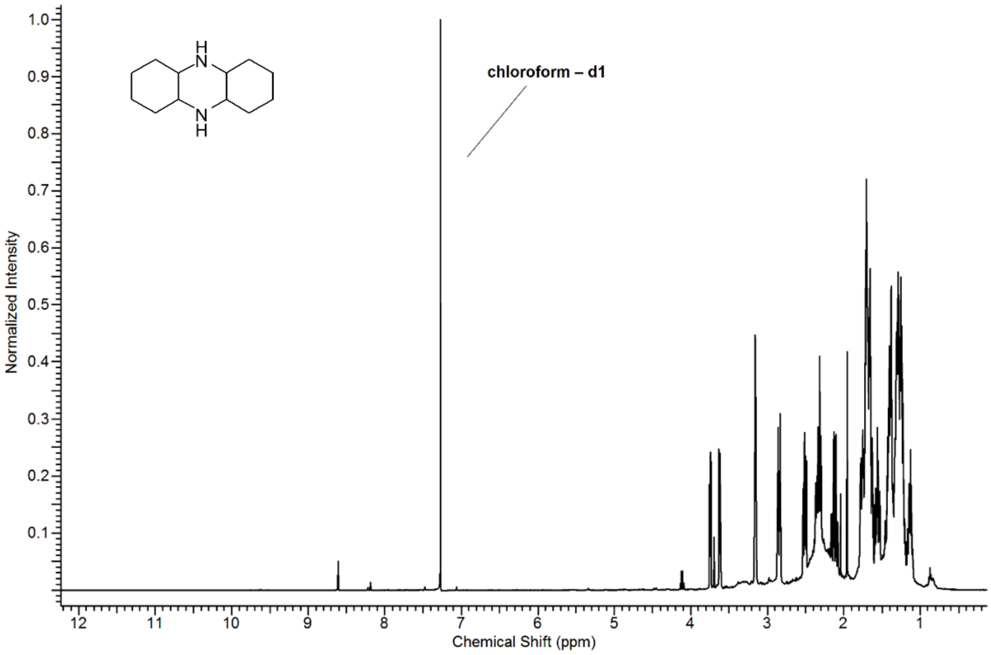

Supplementary Figure 19: ^{1}H-NMR Spectra of tetradecahydrophenazine.

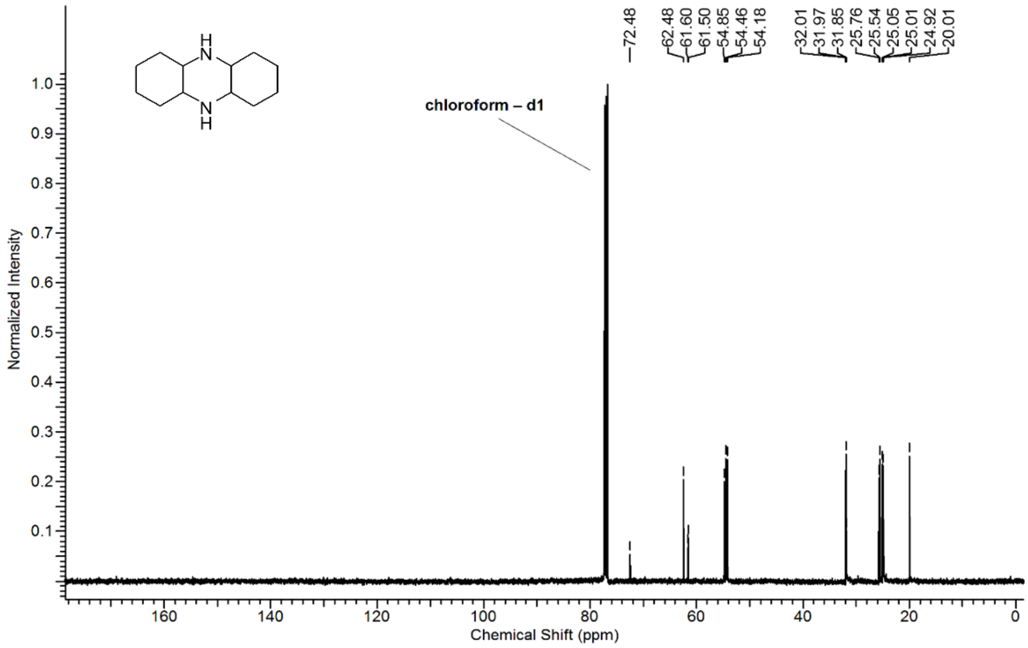

Supplementary Figure 20: ^{13}C-NMR Spectra of tetradecahydrophenazine.

Supplementary Table 1: Optimization of the Pd:Ru for the hydrogenation of NEC and the dehydrogenation of 12H-NEC

**Hydrogenation**			**Yield / %**				
**No.**	**Pd : Ru**	**NEC**	**4H-NEC**	**6H-NEC**	**8H-NEC**	**12H-NEC**	**H_2-Uptake/ wt.-%**
**1**	2 : 1	24	35	2	6	33	2.90
**2**	1 : 1	71	19	1	1	8	0.93
**3**	1 : 2	67	21	1	1	10	1.04
**Dehydrogenation**			**Yields / %**				
**No.**	**Pd : Ru**	**NEC**	**4H-NEC**	**6H-NEC**	**8H-NEC**	**12H-NEC**	**H_2-Release / wt.-%**
**4**	2 : 1	6	45	19	26	4	3.13
**5**	1 : 1	3	10	8	68	11	2.15
**6**	1 : 2	0	10	2	11	77	0.64

Reaction conditions: Hydrogenation of N-ethylcarbazole (NEC): 1 mmol N-ethylcarbazole, 20 mg catalyst (0.26 mol-% active metal), 110 °C, 20 bar H_2 pressure, neat, 36 h. Dehydrogenation of 9-ethyldodecahydro-carbazole (12H-NEC): 2 mmol 12H-NEC, 20 mg catalyst (0.13 mol-% active metal), 172 °C, 1 mL diglyme, 20 h, Ar flow (4-6 mL/min). Yields were determined by GC and GC-MS. H_2-uptake and release was calculated taking all the intermediate products into account; wt.-% of hydrogen is calculated based on the substrate.

Supplementary Table 2: Catalyst screening for the hydrogenation of N-ethylcarbazole (NEC)

		Yield / %[a)]					
**No.**	**Catalyst**	**NEC**	**4H-NEC**	**6H-NEC**	**8H-NEC**	**12H-NEC**	**H_2-Uptake / wt.-%**
**1**	Pd_2Ru@SiCN[b)]	24	35	2	6	33	2.90
**2**	Pd_2Ru@SiCN	0	1	1	1	97	5.68
**3**	Ru@SiCN	0	0	4	3	91	5.53
**4**	Pd@SiCN	100	0	0	0	0	0.00
**5**	Ir@SiCN	90	7	0	2	1	0.29
**6**	Ru/C (5 %)[c)]	59	17	0	12	12	1.49
**7**	Ru/Al_2O_3 (5 %)[c)]	0	0	0	58	42	4.68
**8**	Pd/C (10 %)[c)]	52	30	0	1	17	1.60
**9**	Pd/SiO_2 (5 %)[c)]	100	0	0	0	0	0.00
**10**	Pd/C (10 %) + Ru/C (5%)[d)]	65	20	0	3	12	1.22

Reaction conditions: 1 mmol N-ethylcarbazole, 0.52 mol-% active metal (referring to 40 mg Pd_2Ru@SiCN), 110 °C, 20 bar H_2 pressure, neat, 36 h. Yields were determined by GC and GC-MS. H_2-uptake was calculated taking all the intermediate products into account; wt.-% of hydrogen is calculated based on the substrate. a) Yields were determined by GC and GC-MS. b) 20 mg catalyst (0.26 mol-% active metal); c) The amount of ruthenium was 4.32 µmol referring to the total active metal content of the Pd_2Ru@SiCN catalyst. d) Mixture of 6 mg Pd/C and 6 mg Ru/C (0.17 mol-% active ruthenium and 0.35 mol-% active palladium)

Supplementary Table 3: Catalyst screening for the dehydrogenation of 9-ethyl-dodecahydro-carbazole (12H-NEC)

Yield / %[a]							
**No.**	**Catalyst**	**NEC**	**4H-NEC**	**6H-NEC**	**8H-NEC**	**12H-NEC**	**H_2-Release / wt.-%**
**1**	Pd_2Ru@SiCN[b)]	6	45	19	26	4	3.13
**2**	Pd_2Ru@SiCN	84	16	0	0	0	5.51
**3**	Ru@SiCN	0	0	29	0	71	0.87
**4**	Pd@SiCN	80	18	0	0	2	5.39
**5**	Ir@SiCN	0	1	10	9	80	0.41
**6**	Pd/C (10 %)[c)]	17	69	0	12	2	3.89
**7**	Pd/SiO_2 (5 %)[c)]	0	14	3	21	62	1.03
**8**	Ru/C (5 %)[c)]	0	0	1	3	96	0.03
**9**	Ru/Al_2O_3 (5 %)[c)]	0	0	1	1	98	0.01
**10**	Pd/C (10 %) + Ru/C (5 %)[d)]	18	66	0	13	3	3.85

Reaction conditions: 2 mmol 12H-NEC, 0.52 mol-% active metal (referring to 40 mg Pd_2Ru@SiCN), 180 °C, neat, 7 h, Ar flow (4-6 mL/min). Yields were determined by GC and GC-MS. H_2-release was calculated taking all the intermediate products into account; wt.-% of hydrogen is calculated based on the substrate. a) Yields were determined by GC and GC-MS. b) 20 mg catalyst (0.26 mol-% active metal); c) The amount of palladium was 4.32 µmol referring to the total active metal content of the Pd_2Ru@SiCN catalyst. d) Mixture of 6 mg Pd/C and 6 mg Ru/C (0.17 mol-% active ruthenium and 0.35 mol-% active palladium).

Supplementary Table 4: Catalyst reusability and catalytic H_2 storage with NEC

Yield / %						
**No.**	**NEC**	**4H-NEC**	**6H-NEC**	**8H-NEC**	**12H-NEC**	**H_2-Release/Uptake / wt.-%**
**1-H**	0	0	0	1	99	5.78
**2-De**	98	2	0	0	0	5.74
**3-H**	0	1	0	1	98	5.72
**4-De**	100	0	0	0	0	5.74
**5-H**	0	0	1	3	96	5.71
**6-De**	99	1	0	0	0	5.69

Reaction conditions: 1.0 g (5.12 mmol) N-ethylcarbazole, 200 mg Pd/Ru@SiCN (0.52 mol-% active metal); Hydrogenation: 110 °C, 20 bar H_2, 36 h; Dehydrogenation: 181 °C, Ar flow (4-6 mL/min), 20 h; The hydrogen uptake and release values were calculated by GC and GC-MS analysis. The maximum hydrogen uptake or release is 5.8 wt.-% (calculated based on the substrate). Yields were determined by GC and GC-MS. H_2-uptake or release was calculated taking all the intermediate products into account. The H_2-storage or release values are based on the H_2-storage or release of the former step.

Supplementary Table 5: Catalyst reusability and reversible hydrogen storage with phenazine

Yield / %					
**No.**	**Phen**	**4H-Phen**	**8H-Phen**	**14H-Phen**	**H_2-Release/Uptake / wt.-%**
**1-H**	0	0	15	85	6.77
**1-De**	100	0	0	0	6.77
**2-H**	0	0	5	95	7.05
**2-De**	94	6	0	0	6.94
**3-H**	0	4	17	79	6.35
**3-De**	99	1	0	0	6.42
**4-H**	0	0	9	91	6.85
**4-De**	77	0	23	0	5.81
**5-H**	0	0	5	95	5.93
**5-De**	93	0	7	0	6.70
**6-H**	0	0	6	94	6.66
**6-De**	99	1	0	0	7.01
**7-H**	0	1	3	96	7.08
**7-De**	90	0	10	0	6.56

Reaction conditions: 360 mg (2 mmol) phenazine, 70 mg Pd_2Ru@SiCN (0.46 mol-% active metal); Hydrogenation: 115 °C, 50 bar H_2, 2 mL dioxane, 0.5 mL water, 24 h; Dehydrogenation: 190 °C, 0.75 mL digylme, slight Ar flow (4-6 mL/min). The maximum hydrogen uptake or release is 7.2 wt.-% (calculated based on the substrate).The hydrogen uptake and release values were calculated by GC and GC-MS analysis. Yields were determined by GC and GC-MS. H_2-storage or release was calculated taking all the intermediate products into account. The H_2-storage or release values are based on the H_2-storage or release of the former step.

Supplementary Note 1

Different diastereoisomeres lead to an overlay of the signals and prohibit a clear spectroscopic analysis of tetradecahydrophenazine. However, the lack of aromatic signals is distinctive. Additionally high resolution mass spectroscopy was accomplished.
HRMS (ESI): calcd. for $C_{12}H_{23}N_2$ $[M+H]^+$: 195.18558; found: 195.18504.

Supplementary Methods

General Methods

All reactions were carried out in a dry argon or nitrogen atmosphere using standard Schlenk or glove box techniques. Halogenated solvents were dried over P_2O_5, and nonhalogenated solvents were dried over sodium benzophenone ketyl. All chemicals were purchased from commercial sources with purity over 95 % and used without further purification. Polysilazane "KiON HTT1800" was purchased from Clariant Advanced Materials GmbH, Frankfurt (Germany) and used without further purification. NMR spectra were received using an INOVA 300 MHz spectrometer at 298 K. Chemical shifts are reported in ppm relative to the deuterated solvent. The STEM-EDX analysis was performed at a FEI Titan G2 80-200 S/TEM equipped with a Super-X EDX system, operating at 200 kV, at "Bayerisches Geoinstitut" (University of Bayreuth). Elemental analyses were carried out on a Vario elementar EL *III*. X-ray crystal structure analyses were performed with a STOE-STADIVARI diffractometer [λ(Mo-K_α) = 0.71073 Å] equipped with an Oxford Cryostream low-temperature unit. Structure solution and refinement were accomplished with SIR-97 and SHELXL-201[1,2]. Ceramization was carried out under nitrogen atmosphere in a high temperature furnace (Gero, Germany). GC analyses were carried out on an Agilent 6890N Network GC system equipped with a HP-5 column (30 m x 0.32 µm x 0.25 µm) using *n*-dodecane as internal standard. GC/MS analyses were carried out on an Agilent 7890A/MSD 5975C system equipped with a HP-5MS column (30 m x 0.32 µm x 0.25 µm).

Synthesis and Characterization of the Pd_2Ru@SiCN Catalyst

The bis(2-methylallyl)(1,5-cyclooctadiene)ruthenium(II) complex was purchased from the company "Sigma Aldrich" and used without further purification. 4-Methyl-2-((trimethylsilyl)-amino)pyridine $Ap^{TMS}H$ was synthesized according to a published procedure in 92 % yield[3]. The palladium complex **2** was synthesized according to a published procedure in 53 % yield[4].

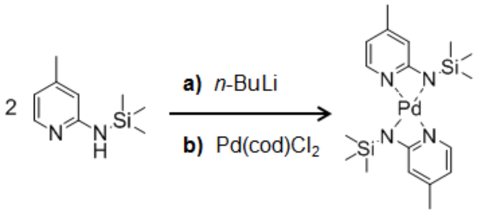

$Ap^{TMS}H$ **$Pd(Ap^{TMS})_2$**

Under vigorous stirring 173 mg HTT1800 was added drop wise to a solution of 84 mg (0.18 mmol) [$PdAp^{TMS}{}_2$], 29 mg (0.09 mmol) bis(2-methylallyl)(1,5-cyclooctadiene)-ruthenium(II) and 5 mg dicumylperoxid (2.9 wt.-%) in 1.5 mL thf. The reaction vial was immediately placed in a pre-heated oilbath at 120 °C for 24 h. After cooling down the solvent was removed under reduced pressure and the brown-black solid was pyrolyzed under N_2 atmosphere with the following heating program:

$$25\ °C \xrightarrow{1\ °C/min} 300\ °C\ (1\ h) \xrightarrow{5\ °C/min} 750\ °C\ (1\ h) \xrightarrow{4\ °C/min} 25\ °C$$

The ceramic yield was 77 %. After ball milling for 15 minutes ("Pulverisette 0"; Fritsch, Germany), the catalyst was pre-treated by stirring in an aqueous solution of NaOH (c = 1 mol/l) at 60 °C for 12 h.

ICP-OES-Analysis

ICP-OES measurements were carried out by using a Vista-pro radical model from Varian. 50 mg of the sample was solved in 1.5 mL HNO_3 (65 %, distilled), 4.5 mL HCl (32 %, p.a.) and 1 mL HF (40 %) and heated in the microwave at 170 °C for 7 min (80 % power), at 180 °C for 7 min (85 % power) and at 195 °C for 20 min (90 % power).

Result: 7.56 wt.-% Pd content

3.95 wt.-% Ru content

The metal contents are slightly lower than expected (8.92 wt.-% Pd, 4.26 wt.-% Ru).

Hydrogen Chemisorption Measurements

Hydrogen chemiesorption measurements were carried out by using a ChemBET Pulsar TPR/TPD instrument from Quantachrome. 30 mg of the Pd_2Ru@SiCN catalyst were pre-treated under helium and nitrogen atmosphere at 400 °C for 3 hours. After cooling the sample to RT, hydrogen gas (5 % in N_2) was added portion wise using a 250 µL injection loop. Analysis of the results was performed with the free version of the program fytik 0.9.8[5]. The metal content of the sample was calculated to be 8.92 wt.-% palladium and 4.26 wt.-% ruthenium. Metal dispersion on the surface of Pd_2Ru@SiCN catalyst (The main component palladium was chosen for calculation): 8.57 %

N_2 Sorption Measurements

N_2 sorption measurements were carried out using a Nova2000e (Quantachrome). The BET surface area was calculated to be 82 m^2/g (Supplementary Fig. 1). The NLDFT equilibrium model (N_2 at 77 K) with slit/cylindrical pores on Carbon surface was chosen due to the lowest fitting error.

Powder XRD analysis

X-ray powder diffractograms were recorded by using a STOE STADI-P-diffractometer (CuK_α-radiation, 1.54178 Å) in θ-2θ-geometry and with a position sensitive detector. The result of the powder XRD analysis of the Pd_2Ru@SiCN catalyst is shown in Supplementary Fig. 2. A median particle diameter of 1.6 nm was calculated by Debye-Scherrer-equation.

Size of the Pd Nanoparticles

The size of the palladium nanoparticles were measured with the programm “ImageJ”. The median particle diameter was measured to be 1.6 nm (Supplementary Fig. 3).

TPR Measurements

Temperature programmed reduction (TPR) measurements were performed using a ChemBET Pulsar TPR/TPD instrument from Quantachrome. 12 mg of the sample were oxidized on air by heating with 5 K/min to 500 °C for 3 h. Afterwards the sample was transferred to an u-tube and pre-treated under nitrogen atmosphere at 200 °C for 0.5 h. The sample was cooled down under nitrogen atmosphere and flushed with hydrogen (5 % in N_2). TPR analysis was performed by heating the sample with 5 K/min to 525 °C and the results were analyzed using the program fytik 0.9.8[5] (Supplementary Fig. 4).

Synthesis and Characterization of the Ir@SiCN Catalyst

The used Ir@SiCN catalyst was synthesized, characterized and used as reported.[6]

Synthesis and Characterization of the Ru@SiCN Catalyst

The bis(2-methylallyl)(1,5-cyclooctadiene)ruthenium(II) complex was purchased from the company Sigma Aldrich and used without further purification.

Under vigorous stirring 0.552 g (638 µL) HTT1800 was added drop wise to a solution of 274 mg (0.86 mmol) bis(2-methylallyl)(1,5-cyclooctadiene)ruthenium(II) and 15 mg dicumylperoxid (2.7 wt.-%) in 6 mL thf. Half of the solvent was removed slowly under reduced pressure and the resulting solution was crosslinked at 120 °C for 24 h. After removal of the solvent the brown-black solid was pyrolyzed under N_2 atmosphere with the following heating program:

$$25\ °C \xrightarrow{1\ °C/min} 300\ °C\ (1\ h) \xrightarrow{5\ °C/min} 900\ °C\ (1\ h) \xrightarrow{4\ °C/min} 25\ °C$$

The ceramic yield was 81 %. After ball milling for 15 minutes ("Pulverisette 0"; Fritsch, Germany), the catalyst was pre-treated by stirring in an aqueous solution of NaOH (c = 1 mol/l) at 60 °C for 12 h.

ICP-OES Analysis

ICP-OES measurements were carried out by using a Vista-pro radical model from Varian. 25 mg of the sample was solved in 1.5 mL HNO_3 (65 %, distilled), 4.5 mL HCl (32 %, p.a.) and 1 mL HF (40 %) and heated in the microwave at 170 °C for 7 min (80 % power), at 180 °C for 7 min (85 % power) and at 195 °C for 20 min (90 % power).

Result: 9.68 wt% Ru content.

Hydrogen Chemiesorption Measurement

Hydrogen chemiesorption measurements were carried out by using a ChemBET Pulsar TPR/TPD instrument from Quantachrome. 59 mg of the Ru@SiCN catalyst was pre-treated under helium and nitrogen atmosphere at 450 °C for 3 hours. After cooling the sample to RT, hydrogen gas was added portion wise using a 50 µL injection loop. Analysis of the results was performed with the free version of the program fytik 0.9.8.[5]
Metal dispersion on the surface of Ru@SiCN catalyst: 6.16 %.

N_2 Sorption

N_2 sorption measurements were carried out using a Nova2000e (Quantachrome).The BET surface area was calculated to be 208 m^2/g (Supplementary Fig. 5). The NLDFT equilibrium model (N_2 at 77 K) with slit/cylindrical pores on Carbon surface was chosen due to the lowest fitting error.

Powder XRD Analysis

X-ray powder diffractograms were recorded by using a STOE STADI-P-diffractometer (CuK_α-radiation, 1.54178 Å) in θ-2θ-geometry and with a position sensitive detector. The result of the powder XRD analysis is shown in Supplementary Fig. 6. The size of the Ru nanoparticles was calculated to be 0.9-1.1 nm by Debye-Scherrer-equation.

HR-TEM Analysis

High-resolution transmission electron microscopy (HR-TEM) was carried out by using a Philips CM300 FEG/UT (300 kV) instrument. The sample was suspended in chloroform and sonicated for 2 min. Subsequently a drop of the suspended sample was placed on a grid with lacy carbon film and allowed to dry. The Ru@SiCN catalyst was analyzed by TEM and HR-TEM to reveal the nature of the particles (Supplementary Fig. 7). Analysis of the FFT resulted in a d-spacing of 205.6 ± 1.7 pm is in accordance with the theoretical value of 204.9 pm for the (101)-reflex of hexagonal crystalline ruthenium.

Size of the Ru Nanoparticles

The size of the ruthenium nanoparticles were measured with the program "ImageJ". The median particle diameter was calculated to be 0.93 nm (Supplementary Fig. 8).

Synthesis and Characterization of the Pd@SiCN Catalyst

Under vigorous stirring 183 mg HTT1800 was added drop wise to a solution of 66 mg (0.14 mmol) $Pd(Ap^{TMS})_2$ and 5 mg dicumylperoxid (2.7 wt.-%) in 1 mL thf. The reaction vial was immediately placed in a pre-heated oil bath at 110 °C for 24 h. After cooling down the solvent was removed under reduced pressure and the brown-black solid was pyrolyzed under N_2 atmosphere with the following heating program:

$$25\ ^\circ C \xrightarrow{1\ ^\circ C/min} 300\ ^\circ C\ (1\ h) \xrightarrow{5\ ^\circ C/min} 750\ ^\circ C\ (1\ h) \xrightarrow{4\ ^\circ C/min} 25\ ^\circ C$$

The ceramic yield was 78 %. After ball milling for 15 minutes ("Pulverisette 0"; Fritsch, Germany), the catalyst was pre-treated by stirring in an aqueous solution of NaOH (c = 1 mol/l) at 60 °C for 12 h.

ICP-OES Analysis

ICP-OES measurements were carried out by using a Vista-pro radical model from Varian. 50 mg of the sample was solved in 1.5 mL HNO_3 (65 %, distilled), 4.5 mL HCl (32 %, p.a.) and 1 mL HF (40 %) and heated in the microwave at 170 °C for 7 min (80 % power), at 180 °C for 7 min (85 % power) and at 195 °C for 20 min (90 % power).

Result: 8.31 wt.-% Pd content

Hydrogen Chemisorption Measurement

Hydrogen chemisorption measurements were carried out by using a ChemBET Pulsar TPR/TPD instrument from Quantachrome. 90 mg of the Pd@SiCN catalyst was pre-treated under helium and nitrogen atmosphere at 400 °C for 3 hours. After cooling the sample to RT, hydrogen gas (5 % in N_2) was added portion wise using a 250 µL injection loop. Analysis of the results was performed with the free version of the program fytik 0.9.8.[5]

Metal dispersion on the surface of Pd@SiCN catalyst: 4.60 %.

N_2 Sorption

N_2 sorption measurements were carried out using a Nova2000e (Quantachrome). Experiments revealed no porosity of the Pd@SiCN nanocomposite as synthesized.

Powder XRD Analysis

X-ray powder diffractograms were recorded by using a STOE STADI-P-diffractometer (CuK_{α}-radiation, 1.54178 Å) in θ-2θ-geometry and with a position sensitive detector. The 2θ values of 40.1°, 46.3° and 67.8° can be assigned to the (111), (200) and (220) reflexes of cubic crystalline palladium (Supplementary Fig. 9).

TEM Analysis

Transmission electron microscopy (TEM) was carried out by using a Varian LEO 9220 (200 kV) instrument. The sample was suspended in chloroform and sonicated for 5 min. Subsequently a drop of the suspended sample was placed on a grid (Plano S 166-3) and allowed to dry. The Pd nanoparticles are homogenously distributed (Supplementary Fig. 10) and the mean particle diameter, determined by the program "Image J," is 1.8 nm (Supplementary Fig. 11).

Catalyst Screening

For our catalytic reactions, we use the following nomenclature for the intermediate products:

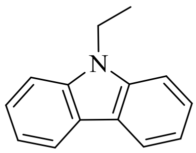	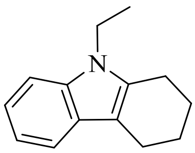	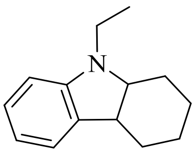	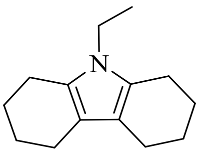	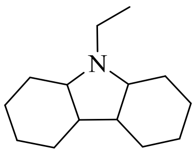
NEC	4H-NEC	6H-NEC	8H-NEC	12H-NEC

The maximum hydrogen uptake or hydrogen release for the NEC/12H-NEC system is 5.8 wt.%. For all examples, the wt.-% is calculated based on the substrate, not taking in consideration the solvent. All H_2-uptake and H_2-release values were calculated by GC and GC-MS taking all the intermediate products into account. Dodecahydro-N-ethylcarbazole (12H-NEC) was synthesized on large scale according to published procedure.[7]

Hydrogenation of NEC

In a typical procedure, the catalyst and NEC are filled in a glass reaction vial which is placed in 250 mL stainless steel autoclave. The autoclave is flushed with hydrogen gas three times, pressured with hydrogen gas and then heated to the desired temperature. After the reaction time, the autoclave was placed in an ice bath, the hydrogen pressure was released and a sample for GC and GC-MS analysis was taken.

Dehydrogenation of 12H-NEC

In a typical procedure the catalyst and 12H-NEC are filled in a 10 mL Schlenk tube and the mixture was evacuated and flushed with argon three times. The tube was placed in a pre-heated oil bath and a slight argon flow of 4-6 mL/min was adjusted. After the desired reaction time the tube was cooled to RT under argon atmosphere and a sample for GC and GC-MS analysis was taken.

Optimization of Pd : Ru Ratio

The Ru : Pd ratio was optimized in the hydrogenation of NEC and dehydrogenation of 12H-NEC. For the Hydrogenation reaction, 1 mmol NEC, 20 mg catalyst (0.26 mol-% active metal) and a magnetic stirring bar were given in a reaction vial and the vial was placed in a 250 ml stainless steel autoclave. After flushing three times with 30 bar hydrogen the autoclave was pressured to 20 bar hydrogen and the reaction took place within 36 hours at 110 °C. After the reaction time, the autoclave was placed in an ice bath, the hydrogen pressure was released and a sample for GC and GC-MS analysis was taken. For the dehydrogenation reaction, 2 mmol 12H-NEC, 20 mg catalyst (0.13 mol-% active metal) and 1 ml diglyme were given in a 10 ml schlenk tube. The mixture was evacuated and flushed with argon three times. The tube was placed in a pre-heated oil bath and a slight argon flow of 4-6 mL/min was adjusted. After the desired reaction time the tube was cooled to RT under argon atmosphere and a sample for GC and GC-MS analysis was taken. (Supplementary Table 1). H_2-storage was calculated taking all the intermediate products into account and the wt.-% of hydrogen is calculated based on the substrate (Supplementary Table 1).

40 mg of the designed Pd_2Ru@SiCN catalyst contains 2.8 µmol active palladium and 1.44 µmol active ruthenium on the surface. So we tested the commercial catalysts in the dehydrogenation of 12H-NEC with the particular amounts of palladium or ruthenium or mixtures of them. (For a detailed evaluation of the results please see (Supplementary Table 2, Supplementary Table 3).

Reversible Hydrogen Storage and Catalyst Reusability with the NEC System

Hydrogenation: 1.0 g (5.12 mmol) NEC and 200 mg Pd_2Ru@SiCN (0.52 mol-% active metal) were given in a 250 mL stainless steel autoclave and flushed with hydrogen three times. A hydrogen pressure of 20 bar was adjusted and the reactor was heated to 110 °C for 36 h. After cooling to room temperature with an ice bath, a sample for GC and GC-MS was taken and the mixture was transferred to a 40 mL Schlenk tube for dehydrogenation.

Dehydrogenation: The Schlenk tube was evacuated and flushed with argon three times and placed in a preheated oil bath at 190 °C. The reaction temperature was measured to be 180 °C. A slight argon flow of 4-6 mL/min was adjusted. After 20 h the mixture was cooled to room temperature and a sample for by GC and GC-MS was taken. Afterwards the mixture was again transferred to a 250 mL stainless steel autoclave for hydrogenation.

The exact product distributions can be found in Supplementary Table 4.

Hydrogen Release Experiment

1.25 mmol 12-H NEC and 40 mg Pd_2Ru@SiCN were given in a 10 mL Schlenk tube and the mixture was evacuated and flushed with argon for three times. Afterwards the argon pressure was released and the Schlenk tube was placed in a pre-heated oil bath at 190 °C for 20 h. The hydrogen was collected by a water column.

Result: 157 mL (6.4 mmol) H_2, which refers to 85 % (4.93 wt.%) H_2-release, could be collected. The GC and GC-MS results suggested a H_2 release of 89 % (5.16 wt.%).

To guarantee the purity of the generated hydrogen, the released gas was analyzed by gas chromatography. Therefor a Schlenk tube was prepared as described above, but in this case closed with a septum cap. The mixture was evacuated and backfilled with argon for three times.

Afterwards, the Schlenk tube was placed in a preheated oil bath at 190 °C. The Argon pressure was released and in a continuous interval, gas samples were taken by syringe and analyzed by gas chromatography (Supplementary Fig. 12).

Reversible Hydrogen Storage and Catalyst Reusability with the Phenazine System

For our catalytic reactions we use the following nomenclature for the intermediate products:

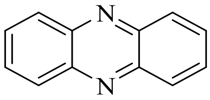	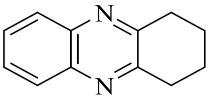	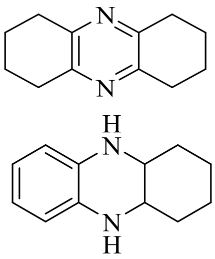	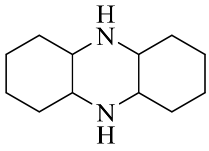
Phen	4H-Phen	8H-Phen	14H-Phen

The maximum hydrogen uptake or hydrogen release for the Phen/14H-Phen system is 7.2 wt.%. For all examples, the wt.-% is calculated based on the substrate, not taking in consideration the solvent. All H_2-uptake and H_2-release values were calculated by GC and GC-MS taking all the intermediate products into account.

Hydrogenation: A reaction vial containing a solution of 360 mg (2 mmol) phenazine and 70 mg Pd_2Ru@SiCN (0.46 mol-% active metal) in 2 mL dioxane and 0.5 mL water was given in a 250 mL stainless steel autoclave. The autoclave was flushed with hydrogen three times and a hydrogen pressure of 50 bar was adjusted. The reactor was heated to 115 °C for 24 h. After cooling to room temperature with an ice bath a sample for GC and GC-MS was taken and the mixture was transferred to a 20 mL Schlenk tube.

Dehydrogenation: The solvents were removed under reduced pressure and 0.75 mL diglyme was added. The tube was evacuated and flushed with argon for three times and a slight argon flow of 4-6 mL/min was adjusted. The tube was placed in a preheated metal bath at 200 °C (190 °C reaction temperature). After 24 h the mixture was cooled to room temperature under argon atmosphere and a sample for GC and GC-MS analysis was taken. Afterwards the mixture was solved in dioxane/water again and transferred to a 250 mL stainless steel autoclave for hydrogenation.

The exact product distributions can be found in Supplementary Table 5.

Hydrogen release experiment

1.25 mmol 14-H Phen and 40 mg Pd_2Ru@SiCN were given in a 10 mL Schlenk tube and the mixture was evacuated and flushed with argon for three times. Afterwards the argon pressure was released and the Schlenk tube was placed in a pre-heated oil bath at 190 °C for 20 h. The hydrogen was collected by a water column.

Result: 186 mL (7.6 mmol) H_2, which refers to 87 % (6.26 wt.%) H_2-release, could be collected. The GC and GC-MS results suggested a H_2 release of 86 % (6.19 wt.%).

To guarantee the purity of the generated hydrogen, the released gas was again analyzed by gas chromatography (Supplementary Fig. 13).

Catalytic Synthesis of Phenazine

Step 1: Reaction of cyclohexane-1,2-diol with ammonia and acceptorless dehydrogenation to 1,2,3,4,6,7,8,9-octahydrophenazine

In a 250 mL stainless steel autoclave 250 mg Ir@SiCN[6] was added to a solution of 2.32 g (20 mmol) cyclohexane-1,2-diol in 12 mL of degassed water. The reactor was flushed three times with ammonia, pressured to 5 bar and the reaction mixture was stirred at 105 °C. After 24 h the ammonia atmosphere was released and the reactor was again pressured with 5 bar ammonia and the reaction mixture was allowed to stir for another 24 h. After cooling to room temperature with an ice bath the mixture was extracted two times with 100 mL diethylether. The organic phase was reduced to the half and extracted with 150 mL of 0.1 M HCl. The water phase was basified with NaOH and extracted two times with 100 mL diethylether. The organic phase was dried over Na_2SO_4 and the solvent was removed giving the light yellow product in 74 % yield.

^{1}H NMR (300 MHz, $CDCl_3$, 298 K): δ = 2.88-2.84 (m, 8H), 1.86-1.90 (m, 8H) ppm. ^{13}C NMR (75 MHz, $CDCl_3$, 298 K): δ = 149.3, 31.6, 22.8 ppm.

elemental analysis (%) for $C_{12}H_{16}N_2$ calcd: C 76.55, H 8.57, N 14.88; found: C 75.54, H 8.54, N 14.16.

Step 2: Acceptorless dehydrogenation to phenazine

A mixture of 25 mg (0.33 mol-% active metal) Pd_2Ru@SiCN catalyst, 0.5 mL digylme and 188 mg (1.0 mmol) 1,2,3,4,6,7,8,9-octahydrophenazine was given in a 10 mL Schlenk tube and evacuated and flushed with argon for two times. The Schlenk tube was placed in a preheated metal bath at 200 °C (190 °C reaction temperature) for 20 h and a slight argon flow of 4-6 mL/min was adjusted. After cooling down under Ar atmosphere the catalyst was separated by centrifugation and washed three times with acetone. The centrifugates were combined and the solvent was removed under reduced pressure at 60 °C giving the yellow crystalline product in 99 % yield.

^{1}H NMR (300 MHz, $CDCl_3$, 298 K): δ = 8.29-8.23 (m, 4H), 7.88-7.82 (m, 4H) ppm. ^{13}C NMR (75 MHz, $CDCl_3$, 298 K): δ = 143.5, 130.5, 129.7 ppm.

Supplementary References

1. Altomare, A., *et al.* SIR97: a new tool for crystal structure determination and refinement. *J. Appl. Crystallogr.* **32**, 115-119, (1999).

2. Sheldrick, G. M. A short history of SHELX, *Acta Crystallogr., Sect. A: Found Crystallogr.* **64**, 112-122, (2008).

3. Kempe, R. & Arndt, P. Mononuclear titanium complexes that contain aminopyridinato ligands. *Inorg. Chem.* **35**, 2644-2649, (1996).

4. Spannenberg, A.; Arndt, P. & Kempe, R. Yttrate-mediated ligand transfer and direct synthesis as a route to amidopalladium complexes. *Angew. Chem., Int. Ed.* **37**, 832-835 (1998).

5. Wojdyr, M. Fityk: a general-purpose peak fitting program. *J. Appl. Cryst.* 43, 1126-1128, (2010).

6. Forberg, D., *et al.* The synthesis of pyrroles via acceptorless dehydrogenative condensation of secondary alcohols and 1,2-amino alcohols mediated by a robust and reusable catalyst based on nanometer-sized iridium particles. *Catal. Sci. Technol.* **4**, 4188-4192 (2014).

7. Eblagon, K. M., *et al.* Hydrogenation of N-ethylcarbazole as prototype of a liquid hydrogen carrier. *Int. J. Hydrogen Energy* **35**, 11609– 11621 (2010).
